# Dental Students’ and Educators’ Perceptions Toward the Adoption of Artificial Intelligence in Dental Education: A Survey‐Based Study

**DOI:** 10.1155/ijod/3313083

**Published:** 2026-07-13

**Authors:** Shimaa Rifaat, Balgis Gaffar, Muhammad Ali Faridi, Faraz Farooqi A., Aisha Alyousif, Taiseer Abdullah Wafai, Rakan Emad Algusier, Noha Taymour

**Affiliations:** ^1^ Department of Restorative Dental Sciences, College of Dentistry, Imam Abdulrahman Bin Faisal University, P.O. Box 1982, Dammam, 31441, Saudi Arabia, iau.edu.sa; ^2^ Department of Preventive Dental Sciences, College of Dentistry, Imam Abdulrahman bin Faisal University, P.O Box 1982, Dammam, 31441, Saudi Arabia, iau.edu.sa; ^3^ Department of Dental Education, College of Dentistry, Imam Abdulrahman Bin Faisal University, P.O. Box 1982, Dammam, 31441, Saudi Arabia, iau.edu.sa; ^4^ College of Dentistry, Imam Abdulrahman bin Faisal University, P.O. Box 1982, Dammam, 31441, Saudi Arabia, iau.edu.sa; ^5^ Department of Substitutive Dental Sciences, College of Dentistry, Imam Abdulrahman Bin Faisal University, P.O. Box 1982, Dammam, 31441, Saudi Arabia, iau.edu.sa

**Keywords:** artificial intelligence (AI), dental curriculum, dental education, dental educators, dental students

## Abstract

**Purpose:**

The integration of artificial intelligence (AI) into dentistry has the potential to transform dental education, clinical training, and research. However, the perspectives of direct stakeholders remain underexplored. This cross‐sectional study aimed to describe current knowledge, perceptions, and self‐reported usage patterns of AI tools among dental students and faculty at a single institution and to identify specific educational needs and barriers to curriculum integration.

**Methodology:**

A cross‐sectional, questionnaire‐based survey was distributed both online and in‐person to dental students and educators. The survey collected demographic data and included closed‐ended items addressing knowledge, perception, and attitudes toward the adoption of AI in dental education. Descriptive statistics and comparative analyses were performed to identify differences across roles, gender, and academic levels.

**Results:**

The study included 333 dental students and 55 faculty members, with a higher proportion of females (53.4%). Most participants were familiar with AI, with significant differences in knowledge levels across academic years (*p* = 0.001). Females reported higher usage of AI for academic tasks (mean = 3.89, *p* = 0.004) and familiarity with AI tools like language translation (mean = 3.49, *p* = 0.009). Faculty showed greater AI knowledge (81.8% vs 27.9%, *p* = 0.001) and were more likely to see its benefits in dental education compared to students.

**Conclusion:**

Both dental students and faculty demonstrated high awareness and positive perceptions regarding AI use and relevance to dental education. The majority expressed strong interest in receiving formal training on AI applications through structured lectures, workshops, and courses. These findings suggest stakeholders’ readiness for AI curriculum integration and indicate a need for curriculum development and establishment of ethical guidelines to support future AI integration, though the extent to which such training would influence educational or clinical outcomes remains to be investigated.

## 1. Introduction

Dental education has evolved through successive waves of digitalization, expanding to social media platforms for professional networking and patient education and now entering a transformative era with artificial intelligence (AI) [1]. AI is an emerging technology in dental education, offering transformative potential in diagnostics, treatment planning, and dental practice management [2]. The integration of AI in dental education must be preceded by a thorough understanding of its functionalities and limitations [3]. While AI is still in its developmental phase, it has already demonstrated its usefulness in diagnostic applications and optimizing clinical workflows [4]. The application of advanced AI models such as ChatGPT and Gemini in dentistry is expanding, particularly in specialized fields [5]. Aziz et al. [6] stated that ChatGPT and Google Gemini can provide reliable responses to inquiries about orthognathic surgery. Similarly, Taymour et al. [4] showed that ChatGPT and Google Gemini provide reliable and useful information in responding to dental implant inquiries. AI chatbots, such as ChatGPT and Gemini, can also improve patient communication, appointment scheduling, and post‐treatment care guidance, making the dental practice more patient‐centric [7]. Despite these advancements, concerns regarding AI reliability, ethical considerations, and the need for human oversight highlight the importance of integrating AI responsibly into clinical workflows [8]. However, its adoption in dental education has received mixed reactions from both educators and students, highlighting the need for an in‐depth assessment of their perceptions [3, 9, 10].

Despite AI’s promising applications, its integration into dental curricula faces several challenges, including a lack of structured training, limited resources, and data availability [11]. Previous studies had indicated that while dental students exhibit a positive attitude towards AI, educators remain hesitant due to concerns regarding their own knowledge gaps and insufficient institutional support [12, 13]. There is also a growing discourse on the ethical implications and potential misuse of AI‐powered tools in academic settings. Furthermore, AI applications long‐term impact on dental training and patient care remains uncertain [14]. This study aimed to describe baseline knowledge, familiarity, and self‐reported usage patterns of AI tools among dental students and faculty; to compare these perceptions across academic levels, gender, and professional roles; and to identify specific educational needs and ethical concerns that could inform future curriculum pilot programs. Findings from this research would help to provide foundational data for evidence‐based AI integration in the dental curriculum, address potential challenges, and inform educational policymakers on the necessary curriculum modifications.

Uniquely, the present study simultaneously surveyed dental students across all academic levels and faculty members from all five departments within the same institutional ecosystem at Imam Abdulrahman Bin Faisal University (IAU), Eastern Province, Saudi Arabia. This within‐institution multi‐stakeholder design eliminates inter‐institutional confounders and enables a more internally valid comparison of AI knowledge, usage, and attitudinal alignment between educators and learners, a design feature that distinguishes this study from prior work that surveys either students or faculty in isolation.

## 2. Materials and Methods

### 2.1. Study Design and Setting

This cross‐sectional survey‐based study employed a convenience sampling strategy with voluntary participation, targeting all eligible dental students and faculty at the college of dentistry, IAU during the 6‐month study period.

### 2.2. Study Participants

The study involved faculty members from all the departments and undergraduate students from 2nd year till 6th year, interns, as well as postgraduate (PG) students. The study included only responders from IAU either faculty members or students with voluntarily completed surveys from online responses or hard copies. Partially completed surveys were excluded.

### 2.3. Data Collection Tool

A validated self‐administered questionnaire (one for faculty and one for students) was distributed through online google forms, scanned QR codes, and hard copies. The questionnaire was adopted from published scientific articles on the research topic [12, 15, 16]. The survey for dental educators consisted of three sections with 28 close‐ended questions in the English language: 4 questions for the demographic data (age, gender, position, and department), and 24 questions about their knowledge about AI, their perception, and attitude with responses (strongly agree, agree, neutral, disagree, and strongly disagree). While the survey for dental students consisted of three sections with 20 close‐ended questions in the English language: two questions for the demographic data (gender, study year), and 18 questions about their AI knowledge, their perception, and attitudes with responses (strongly agree, agree, neutral, disagree, and strongly disagree). To ensure the reliability of the study instrument, a test–retest reliability analysis was conducted on a pilot sample of 30 participants, who completed the questionnaire twice with a 2‐week interval between administrations. The consistency of responses across the two time points was evaluated using the Cohen’s Kappa statistics for each questionnaire item. The reported Kappa value (kappa = 0.87) represents the mean agreement across all items, indicating excellent test–retest reliability. Additionally, the internal consistency of the questionnaire was verified using Cronbach’s alpha (α = 0.789), demonstrating acceptable reliability.

#### 2.3.1. Recruitment Procedures

Potential participants were invited through multiple channels to ensure reachability: (1) email invitations sent to all faculty members and student class representatives; (2) QR codes displayed in common areas of the College of Dentistry (student lounges, clinic areas, and faculty offices); (3) in‐person distribution of paper surveys occurred during regular class hours for students and departmental meetings for faculty; and (4) reminders sent at 2‐week intervals during the data collection period.

To protect anonymity, no identifying information was collected, and paper surveys were deposited in sealed collection boxes. Online responses were tracked only by completion date and survey version (faculty vs. student), with the IP address collection disabled to prevent tracking.

### 2.4. Sample Size Calculation

The sample size calculation was determined using an online software Raosoft with a margin of error of 5%, a confidence interval of 95%, and statistical power at 80%. The sample size was calculated to be 378. To account for potential dropouts or missing data, an additional 10 participants were added, resulting in a total sample size of 388.

### 2.5. Ethical Consideration

All the participants were briefed about the aim of the study, ensuring that their participation is purely voluntary. The survey responses were designed to be anonymous to protect the identity of the participants. Informed consent was obtained from all participants (dental students and faculty members) prior to conducting the survey. Ethical approval was obtained from the Institutional Ethics & Review board – Imam Abdulrahman bin Faisal University (IRB‐2024‐02‐325).

### 2.6. Statistical Analysis

Descriptive statistics were presented as frequencies and percentages for categorical variables and as means ± standard deviations for continuous variables. Categorical variables were analyzed using the Pearson chi‐square test or Fisher’s exact test as appropriate. For comparisons of continuous or ordinal scores between two independent groups, the Mann Whitney *U* test was applied due to non‐normal distribution of the data. Effect size measures were additionally calculated to assess the magnitude of observed differences, including Cohen’s d for continuous variable comparisons and Cramér’s V for categorical associations. Effect sizes were interpreted according to conventional thresholds. All analyses were performed using SPSS (Statistical Package for the Social Sciences), version 28 (IBM Corp., Armonk, NY, USA), and a *p*‐value < 0.05 was considered statistically significant.

## 3. Results

Table 1 summarizes the demographic information of the participants. The sample comprised 388 students and faculty (students = 333 and faculty = 55), with a slightly higher representation of females 207 (53.4%) compared to males 181 (46.6%). Participants were distributed across various academic levels, with the highest percentages being postgraduate students 55 (16.5%), followed by 6th‐year students (53, 15.9%). The distribution among the different faculties was equal for Restorative Dental Sciences (RDS), Substitutive Dental Sciences (SDS), Biomedical Dental Sciences (BDS), and Preventive Dental Sciences (PDS), accounting for 12 (22%).

**Table 1:** Demographical information of the participants.

Demographics		Frequency	Percent
Gender	Male	181	46.6
	Female	207	53.4
Year level	2nd	42	12.6
	3rd	50	15.0
	4th	51	15.3
	5th	45	13.5
	6th	53	15.9
	Intern	37	11.1
	Postgraduate student	55	16.5
Faculty	Restorative Dental Sciences (RDS)	12	22
	Substitutive Dental Sciences (SDS)	12	22
	Biomedical Dental Sciences (BDS)	12	22
	Preventive Dental Sciences (PDS)	12	22
	Dental Education (DE)	7	13

Table 2 shows the knowledge, usage, and familiarity with AI tools among the different study levels and their associations. A majority of participants across all levels have heard about AI. Specifically, 80.5% of undergraduates, 67.5% of interns, and 80% of PG students either agreed or strongly agreed with no statistically significant differences (*p* = 0.088). Among undergraduates, 67.6% agreed or strongly agreed that they had enough information about AI, compared to 83.8% of interns and 56.3% of PG students and the differences were statistically significant (*p* = 0.001). Among the participants, 37% of undergraduates, 30% of interns, and 27% of PG students strongly agreed with the belief that AI could be beneficial in dental education and this difference was statistically significant (*p* = 0.027).

**Table 2:** Participants knowledge, usage, and awareness about the AI.

Knowledge about AI		Students level			*p*‐Value
		Undergraduate *n* = 220	Intern *n* = 37	PG *n* = 55	
I have heard about Artificial Intelligence.	Strongly disagree	4.1%	0.0%	3.6%	0.088
	Disagree	0.0%	0.0%	1.8%	
	Neutral	15.4%	32.4%	14.5%	
	Agree	52.7%	45.9%	47.3%	
	Strongly agree	27.8%	21.6%	32.7%	
I do believe I have enough information and understanding Artificial Intelligence (AI).	Strongly disagree	1.7%	0.0%	0.0%	0.005 ^∗^
	Disagree	11.6%	10.8%	5.5%	
	Neutral	19.1%	5.4%	38.2%	
	Agree	53.5%	64.9%	34.5%	
	Strongly agree	14.1%	18.9%	21.8%	
I think that Artificial Intelligence may be beneficial in dental education.	Strongly disagree	1.7%	0.0%	1.8%	0.027 ^∗^
	Disagree	1.2%	10.8%	3.6%	
	Neutral	14.1%	2.7%	14.5%	
	Agree	46.5%	56.8%	52.7%	
	Strongly agree	36.5%	29.7%	27.3%	
I have been lectured about Artificial Intelligence in my Dental School.	Strongly disagree	0.0%	0.0%	0.0%	0.236
	Disagree	11.6%	2.7%	14.5%	
	Neutral	7.5%	0.0%	7.3%	
	Agree	20.3%	24.3%	23.6%	
	Strongly agree	47.7%	62.2%	36.4%	
I think that AI should be included in the curriculum of dental schools.	Strongly disagree	0.0%	0.0%	0.0%	0.126
	Disagree	1.3%	0.0%	3.6%	
	Neutral	21.3%	24.3%	36.4%	
	Agree	50.8%	40.5%	38.2%	
	Strongly agree	26.7%	35.1%	21.8%	
Usage of AI tools					
I have used AI tools to improve my knowledge about any topic.	Strongly disagree	2.3%	0.0%	7.3%	0.154
	Disagree	2.3%	0.0%	1.8%	
	Neutral	55.5%	64.9%	69.1%	
	Agree	25.9%	24.3%	16.4%	
	Strongly agree	14.1%	10.8%	5.5%	
I have used AI tools to help me finish any of my assignments or assessments in dental school.	Strongly disagree	6.4%	5.4%	12.7%	0.668
	Disagree	4.1%	0.0%	1.8%	
	Neutral	48.2%	51.4%	50.9%	
	Agree	33.6%	37.8%	29.1%	
	Strongly agree	7.7%	5.4%	5.5%	
I am using AI Tools frequently in the following tasks. [Academic Task]	Strongly disagree	3.6%	0.0%	9.1%	0.126
	Disagree	4.1%	0.0%	3.6%	
	Neutral	56.8%	78.4%	58.2%	
	Agree	31.8%	21.6%	23.6%	
	Strongly agree	3.6%	0.0%	5.5%	
I am using AI Tools frequently in the following tasks. [Personal Task]	Strongly disagree	1.8%	2.7%	3.6%	0.034 ^∗^
	Disagree	3.6%	0.0%	7.3%	
	Neutral	50.0%	73.0%	63.6%	
	Agree	32.7%	24.3%	16.4%	
	Strongly agree	11.8%	0.0%	9.1%	
Familiarization of AI tools					
I am familiar with the following AI tools: [Language translation tools]	Strongly disagree	2.3%	0.0%	3.6%	0.114
	Disagree	4.5%	0.0%	3.6%	
	Neutral	54.5%	83.8%	63.6%	
	Agree	25.0%	10.8%	18.2%	
	Strongly agree	13.6%	5.4%	10.9%	
I am familiar with the following AI tools: [Language generation tools]	Strongly disagree	5.0%	8.1%	5.5%	0.033 ^∗^
	Disagree	6.4%	0.0%	9.1%	
	Neutral	57.7%	86.5%	61.8%	
	Agree	20.0%	0.0%	16.4%	
	Strongly agree	10.9%	5.4%	7.3%	
I am familiar with the following AI tools: [Grammar and spell‐checking tools]	Strongly disagree	0.9%	2.7%	1.8%	0.007 ^∗^
	Disagree	4.5%	0.0%	5.5%	
	Neutral	46.8%	81.1%	61.8%	
	Agree	33.2%	16.2%	20.0%	
	Strongly agree	14.5%	0.0%	10.9%	
I am familiar with the following AI tools: [Text summarization tools]	Strongly disagree	0.9%	2.7%	3.6%	0.109
	Disagree	4.1%	0.0%	5.5%	
	Neutral	55.5%	78.4%	58.2%	
	Agree	25.5%	18.9%	21.8%	
	Strongly agree	14.1%	0.0%	10.9%	
I am familiar with the following AI tools: [Text Generators]	Strongly disagree	0.9%	2.7%	3.6%	0.306
	Disagree	4.1%	0.0%	7.3%	
	Neutral	61.8%	78.4%	61.8%	
	Agree	19.5%	13.5%	18.2%	
	Strongly agree	13.6%	5.4%	9.1%	

^∗^Statistically significant at the 0.05 level.

A majority of participants have used AI tools to improve their knowledge on various topics, with 40% of undergraduates, 35.1% of interns, and 21.9% of PG students agreeing or strongly agreeing with no significant differences (*p* = 0.154). However, regarding the usage of AI for personal tasks, the majority of participants remain neutral (50%, 73%, and 64%, respectively), but strong disagreement was noted by 1.8% of undergraduates, 2.7% of interns, and 3.6% of PG students with a statistically significant difference (*p*  = 0.034).

Regarding the familiarity with AI language translation tools, it was higher among undergraduates (38.6%) compared to interns (16.2%) and PG (29.1%) students. Familiarity with language translation tools varied significantly (*p* = 0.033). Among participants, undergraduates (47.7%), compared to 16.2% of interns and 30.9% of PG students, were familiar with grammar and spell‐checking AI tools, a difference that was statistically significant (*p* = 0.007).

Table 3 shows the comparison between male and female students’ knowledge, usage, and familiarization with AI tools. Male students’ knowledge regarding AI was slightly higher. 4.03 ± 0.98 than female students 3.97 ± 0.81 but the difference was not statistically significant (*p* = 0.543). Overall male students showed slightly higher knowledge scores compare to female students but none of the differences were statistically significant (*p*  > 0.05).

**Table 3:** Students’ gender‐wise comparison regarding the knowledge, usage, and familiarization with the AI tools.

Knowledge about AI	Male Mean + SD	Female Mean + SD	*p*‐Value	Cohen’s *d*
I have heard about Artificial Intelligence.	4.03 + 0.98	3.97 + 0.81	0.543	0.07
I do believe I have enough information and understanding Artificial Intelligence (AI).	3.66 + 0.89	3.74 + 0.91	0.416	0.09
I think that Artificial Intelligence may be beneficial in dental education.	4.18 + 0.83	4.06 + 0.85	0.217	0.14
I have been lectured about Artificial Intelligence in my Dental School.	3.47 + 1.1	3.44 + 1.19	0.796	0.03
I think that AI should be included in the curriculum of dental schools.	4.02 + 0.76	3.98 + 0.75	0.651	0.05

**Practice of AI tool**				

I have used AI tools to improve my knowledge about any topic.	3.34 + 0.73	3.46 + 0.91	0.202	0.15
I have used AI tools to help me finish any of my assignments or assessments in dental school.	3.07 + 0.93	3.97 + 0.89	0.001 ^∗^	0.99
I am using AI Tools frequently in the following tasks. [Academic Task]	3.1 + 0.71	3.89 + 0.78	0.004 ^∗^	1.06
I am using AI Tools frequently in the following tasks. [Personal Task]	3.38 + 0.78	3.42 + 0.83	0.680	0.05

**Tools familiar with**				

I am familiar with the following AI tools: [Language translation tools]	3.24 + 0.76	3.49 + 0.87	0.009 ^∗^	0.31
I am familiar with the following AI tools: [Language generation tools]	3.03 + 0.86	3.32 + 0.9	0.004 ^∗^	0.33
I am familiar with the following AI tools: [Grammar and spell‐checking tools]	3.31 + 0.78	3.59 + 0.81	0.003 ^∗^	0.35
I am familiar with the following AI tools: [Text summarization tools]	3.31 + 0.79	3.48 + 0.81	0.059	0.21
I am familiar with the following AI tools: [Text Generators]	3.25 + 0.78	3.43 + 0.82	0.055	0.22

^∗^Statistically significant at the 0.05 level.

However, female students reported significantly higher usage of AI tools for completing assignments (3.97 ± 0.89) (*p* = 0.001) and academic tasks (3.89 ± 0.78) (*p* = 0.004) compared to male students. Female students reported more usage of AI tools for personal tasks (3.42 + 0.83) compared to male students (3.38 + 0.78) but the difference was not statistically significant (*p*  = 0.680). In regard to familiarization with the AI tools, again female students reported significantly higher scores, including language translation (3.49 ± 0.87) (*p* = 0.009), language generation (3.32 ± 0.9) (*p* = 0.004), and grammar and spell‐checking tools (3.59 ± 0.81) (*p* = 0.003) compared to male students. Use of text generation and summarization tools did not show any significant difference between male and female students (*p*  > 0.05). Effect size analysis demonstrated large practical differences for AI usage in assignments (Cohen’s *d* = 0.99) and academic tasks (Cohen’s *d* = 1.06) between male and female students, while familiarity‐related variables showed small‐to‐moderate effect sizes ranging from 0.21 to 0.35.

Table 4 provides a comparison of knowledge, usage, and familiarity with AI tools between students and faculty members. Faculty members reported significantly higher knowledge and understanding of AI (81.8% vs. 27.9%) (*p*  = 0.001). Opinions on whether AI may be beneficial in dental education also varied significantly. With more faculty (3.6%) against AI use in dental education (*p* = 0.044). With regards to the inclusion of AI in the curriculum, a significantly higher proportion of students was in disagreement than faculty (24.1% vs 3.6%) (*p* = 0.001).

**Table 4:** Association between faculty and students’ knowledge, usage, and familiarization about AI.

Knowledge about AI		Comparison		*p*‐Value	Cramer’s *V*
		Students *n* = 333	Faculty *n* = 55		
I have heard about artificial intelligence.	Strongly disagree	3.6%	3.6%	0.001 ^∗^	0.399
	Disagree	0.3%	0.0%		
	Neutral	17.1%	0.0%		
	Agree	51.1%	14.5%		
	Strongly agree	27.9%	81.8%		
I do believe I have enough information and understanding Artificial Intelligence (AI).	Strongly disagree	1.2%	7.3%	0.001 ^∗^	0.254
	Disagree	10.5%	27.3%		
	Neutral	20.7%	25.5%		
	Agree	51.7%	32.7%		
	Strongly agree	15.9%	7.3%		
I think that Artificial Intelligence may be beneficial in dental education.	Strongly disagree	1.5%	3.6%	0.044 ^∗^	0.159
	Disagree	2.7%	0.0%		
	Neutral	12.9%	7.3%		
	Agree	48.6%	36.4%		
	Strongly agree	34.2%	52.7%		
I have been lectured about Artificial Intelligence in my Dental School.	Strongly disagree	11.1%	5.5%	0.001 ^∗^	0.236
	Disagree	6.6%	25.5%		
	Neutral	21.3%	16.4%		
	Agree	47.4%	45.5%		
	Strongly agree	13.5%	7.3%		
I think that AI should be included in the curriculum of dental schools.	Strongly disagree	1.5%	10.9%	0.001 ^∗^	0.264
	Disagree	24.1%	3.6%		
	Neutral	47.6%	63.6%		
	Agree	26.8%	21.8%		
	Strongly agree	1.5%	10.9%		
Usage of AI tool					—
I have used AI tools to improve my knowledge about any topic.	Strongly disagree	2.9%	3.6%	0.001 ^∗^	0.425
	Disagree	1.9%	25.5%		
	Neutral	59.0%	21.8%		
	Agree	24.0%	41.8%		
	Strongly agree	12.2%	7.3%		
I have used AI tools to help me finish any of my assignments or assessments in dental school.	Strongly disagree	7.4%	10.9%	0.001 ^∗^	0.510
	Disagree	3.2%	43.6%		
	Neutral	49.0%	29.1%		
	Agree	33.3%	16.4%		
	Strongly agree	7.1%	0.0%		
I am using AI Tools frequently in the following tasks. [Academic Task]	Strongly disagree	2.2%	3.6%	0.001 ^∗^	0.501
	Disagree	3.8%	25.5%		
	Neutral	55.1%	12.7%		
	Agree	28.8%	49.1%		
	Strongly agree	9.9%	9.1%		
I am using AI Tools frequently in the following tasks. [Personal Task]	Strongly disagree	4.2%	5.5%	0.001 ^∗^	0.385
	Disagree	3.5%	43.6%		
	Neutral	59.6%	29.1%		
	Agree	29.2%	14.5%		
	Strongly agree	3.5%	7.3%		
Tools Familiar with					—
I am familiar with the following AI tools: [Language translation tools]	Strongly disagree	2.2%	3.6%	0.001 ^∗^	0.371
	Disagree	3.8%	25.5%		
	Neutral	59.6%	18.2%		
	Agree	22.1%	34.5%		
	Strongly agree	12.2%	18.2%		
I am familiar with the following AI tools: [Language generation tools]	Strongly disagree	5.4%	3.6%	0.001 ^∗^	0.369
	Disagree	6.1%	25.5%		
	Neutral	61.9%	18.2%		
	Agree	17.0%	41.8%		
	Strongly agree	9.6%	10.9%		
I am familiar with the following AI tools: [Grammar and spell‐checking tools]	Strongly disagree	1.3%	3.6%	0.001 ^∗^	0.385
	Disagree	4.2%	21.8%		
	Neutral	53.5%	12.7%		
	Agree	28.8%	27.3%		
	Strongly agree	12.2%	34.5%		
I am familiar with the following AI tools: [Text summarization tools]	Strongly disagree	1.6%	5.5%	0.001 ^∗^	0.293
	Disagree	3.8%	20.0%		
	Neutral	58.7%	29.1%		
	Agree	24.0%	32.7%		
	Strongly agree	11.9%	12.7%		
I am familiar with the following AI tools: [Text Generators]	Strongly disagree	1.6%	9.1%	0.001 ^∗^	0.303
	Disagree	4.2%	18.2%		
	Neutral	63.8%	32.7%		
	Agree	18.6%	21.8%		
	Strongly agree	11.9%	18.2%		

^∗^Statistically significant at the 0.05 level.

Faculty members reported higher engagement with AI for improving knowledge, with 41.8% agreeing compared to 24.0% of students (*p* = 0.001). However, students showed more frequent usage of AI tools for completing assignments, with 33.3% agreeing, in contrast to only 16.4% of faculty. Faculty were more likely to disagree with using AI tools for assignments (43.6%) compared to students (3.2%). When examining frequent usage for academic tasks, 49.1% of faculty agreed, surpassing the 28.8% of students who reported similar usage (*p* = 0.001). Faculty demonstrated greater familiarity with AI tools compared to students, with significant differences observed across various types of tools (*p* = 0.001). Effect size analysis demonstrated predominantly moderate‐to‐strong associations between participant roles and AI‐related knowledge, usage, and familiarity variables, with the strongest associations observed for AI usage in assignments (Cramér’s *V* = 0.510) and academic tasks (Cramér’s *V* = 0.501).

Table 5 captures the suggestions of both students and faculty regarding incorporating AI tools into dental education. A significant majority (62.6%) believed training on AI tools is necessary for dental students, with faculty (4.76 ± 0.51) more strongly in favor than students (3.74 ± 0.81) (*p* = 0.001). Similarly, 66.8% believed AI will improve the quality and performance of dental students, with faculty (4.44 ± 0.57) more optimistic than students (3.86 ± 0.78) (*p* = 0.001). Concerns about ethical violations due to AI in dental education are noted by 53.4% of participants, with faculty expressing higher concerns (3.96 ± 1.04) compared to students (3.62 ± 0.85) (*p* = 0.007). Effect size estimates demonstrated a very large effect for support toward formal AI training (Cohen’s *d* = 1.51), while perceptions regarding AI improving student performance showed a large effect size (*d* = 0.85). Ethical concerns demonstrated a small‐to‐moderate practical effect (*d* = 0.36).

**Table 5:** Suggestions from faculty and students regarding the inclusion of AI tools in teaching.

Suggestions for AI	Dental students	Faculty	*p*‐Value	Cohen’s *d*
Training should be given about the usage of AI tools for all dental students.	3.74 + 0.81	4.76 + 0.51	0.001 ^∗^	1.51
I do believe that artificial intelligence may improve the quality and performance of dental students in the future.	3.86 + 0.78	4.44 + 0.57	0.001 ^∗^	0.85
I think the usage of AI in the field of dental education can violate ethical principles.	3.62 + 0.85	3.96 + 1.04	0.007 ^∗^	0.36

^∗^Statistically significant at 0.05.

**Figure 1:**
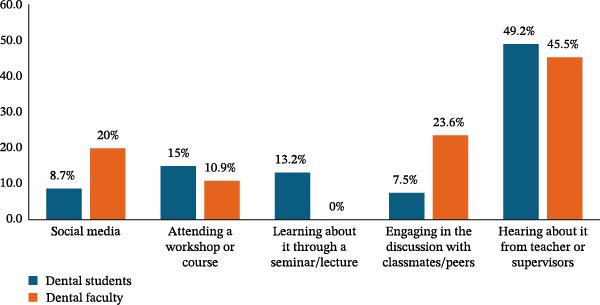
Source of artificial intelligence awareness among dental students and dental faculty.

Figure 1 illustrates the sources of information regarding AI tools between dental students and their faculty. The majority of students (49.2%) reported that they had heard about AI primarily from their teachers or supervisors. Similarly, faculty members also identified teachers or supervisors as their main source of information. Faculty members, however, reported utilization of social media (20%) compared to dental students (8.7%) for acquiring information about AI. Additionally, faculty were more likely to rely on peers (24%) compared to dental students (8%) as a source of AI‐related knowledge.

## 4. Discussion

The present study explored the knowledge, perceptions, and usage of AI among dental students and faculty members. Overall, the findings indicate a generally positive attitude toward AI, with a high level of awareness among participants and significant differences across academic levels, genders, and professional roles. These findings highlight the growing relevance of AI in dental education and the need for structured strategies to support its ethical integration into the dental curricula.

### 4.1. Knowledge and Awareness of AI

In the present study, a large proportion of participants (81.5%) reported the awareness of AI tools and their applications. This finding is consistent with studies conducted in Syria (70%) and India (70%–78.9%) [17, 18] but higher than reports from Turkey (44.8%) and South Korea (24.2%) [19, 20]. However, contrary to the findings of a local study from the capital city Riyadh, 50.1% of their participants had no basic knowledge about the working principle of AI [21]. The observed global increase in AI awareness among dental students is likely due to the rapid expansion of digital technologies in education and clinical practice, as there are many AI tools that can facilitate students’ personal and practical life; therefore, awareness about such tools is valid and essential. However, despite the high awareness in the current study, significant differences in perceived knowledge were observed across academic levels (*p*  < 0.05), indicating that exposure to AI concepts remains inconsistent throughout dental training.

On the other hand, no statistically significant difference was observed between males and females regarding AI knowledge, like the findings by Jeong et al. [20] and Aboalshamat [22]. Contemporary dental education provides the same curriculum and resources to all students, including AI knowledge [23]. AI in dentistry in the same context focuses on using AI diagnostic systems such as digital radiography and CAD/CAM rather than the creation and advancement of AI systems [24]. This practical approach helps reduce gender disparities in AI among dental students while offering equal opportunities for both male and female students to learn.

### 4.2. Usage and Familiarity With AI Tools

The findings of the current study showed that AI tools are commonly used for academic purposes, and there were statistically significant differences between students across the study years, with more familiarization with language generation tools and grammar and spell‐checking tools (40%) reported by undergraduates. These findings suggest that AI tools are already embedded in students’ learning practices. Similar trends have been reported in previous studies, highlighting the increasing reliance on AI‐powered tools for academic tasks [25, 26]. This widespread usage, however, underscores the need for proper guidance to ensure appropriate and ethical use. A contradictory finding was reported by Roganović et al. [27], who found that a lack of working experience was significantly associated with a lack of knowledge regarding AI use in medicine and dentistry. The finding of the current study that junior dental students exhibit a higher level of familiarity with AI tools when compared to seniors may appear contrary to expectations, given that senior students generally possess more extensive clinical and theoretical knowledge. The discrepancy can be attributed to various factors, such as changes in curriculum, advancements in technology, and increased exposure opportunities [28]. While this finding highlights the benefits of early exposure to AI, it underscores the need for institutions to provide consistent and equitable opportunities for all students to engage with emerging technologies. The higher AI tool familiarity observed among junior undergraduate students compared to interns and postgraduate students may reflect a cohort effect rooted in digital‐native generational exposure, whereby students who entered the program more recently were socialized into digital tool use from an earlier age, a pattern consistent with the literature on generational technology adoption in health professions education.

In the same context, female students demonstrated significantly higher usage of AI tools for assignments, academic tasks, along with greater familiarity with language translation, language generation and grammar‐checking tools (*p*  < 0.05). The greater use of AI tools among females for academic purposes could be due to their focus on tasks and the desire for linguistic accuracy. However, there were similarities in how both genders used AI tools outside of academics [29]. Tailored training and awareness programs can improve AI use among students and encourage student AI interest [26]. Mutual agreements are needed between college administration, the curriculum committee, instructors, and program directors for potential curriculum changes to include AI [30]. Although AI has useful applications in dental education, the risk of misuse may lead faculty to be hesitant about its inclusion in dental curricula [31]. This pattern is consistent with the Technology Acceptance Model (TAM), which posits that perceived usefulness, rather than general technological curiosity, is a primary driver of adoption; female students’ higher engagement with task‐oriented tools (assignment completion, language generation, and grammar‐checking) suggests that utility‐driven motivation may underlie this gender‐based usage disparity, despite equivalent knowledge levels between genders [32].

### 4.3. Students vs. Faculty Perspectives

The comparison between students and the faculty revealed important differences. Faculty members demonstrated significantly higher knowledge of AI (81.8% vs. 27.9%, *p*  < 0.05), while students reported more frequent use of AI tools, particularly for assignments (33.3% vs. 16.4%). Faculty were also more likely to disagree with using AI for assignments (43.6% vs. 3.2%, *p*  < 0.05). These findings suggest a gap between theoretical understanding and practical usage. Additionally, faculty expressed greater concern regarding ethical implications (*p* = 0.007), which is consistent with previous studies highlighting concerns about academic integrity and misuse of AI tools [33]. This discrepancy emphasizes the need for alignment between faculty guidance and student practices.

This divergence between faculty’s superior theoretical knowledge and students’ higher practical AI usage exemplifies the “knowing‐doing gap,” a well‐documented phenomenon in educational research whereby theoretical knowledge does not automatically translate into behavioral adoption, particularly in institutional settings where structural support, role expectations, and professional norms may inhibit faculty uptake of emerging digital tools [34]. Addressing this gap requires targeted faculty development initiatives that bridge conceptual AI literacy with practical, discipline‐specific application skills.

In the current study, a significantly higher number of faculty perceived that training should be given about the usage of AI tools for all dental students and believed that AI may improve the quality and performance of dental students in the future. This observed belief may be due to the need to prepare students for their future careers. A dental education review highlighted the increasing use of AI tools and emphasized the need to train students and educators to enhance the effectiveness and reliability of these tools [35]. It is a widespread belief among faculty members that AI has the potential to greatly improve the quality of education and student outcomes. AI applications such as automated grading systems, virtual patient simulations, and personalized learning platforms have shown effectiveness in enhancing students’ practical and decision‐making skills [3].

### 4.4. Educational Implications of AI Integration

The variation in knowledge and usage observed in this study supports the need for structured curriculum development. A majority of participants (62.6%) agreed that AI training is necessary, with faculty showing stronger agreement (mean = 4.76 vs. 3.74, *p*  < 0.05). Similarly, 66.8% believed that AI could improve student performance. These findings align with previous studies advocating for the inclusion of AI in dental curricula [35, 36]. The findings of this study support the need for structured integration of AI into dental curricula. This may include dedicated modules, workshops, or blended learning approaches focusing on AI applications in dentistry. Clearly defined learning outcomes should address knowledge (understanding AI principles), skills (using AI tools), and attitudes (ethical and responsible use). Additionally, incorporating hands‐on training and case‐based learning involving AI tools can further strengthen students’ competence and confidence in this domain. Notably, academic level (*p* = 0.001), gender (*p* = 0.004), and role; student vs. faculty (*p*  < 0.001) emerged as significant predictors of AI knowledge and usage outcomes, providing a preliminary predictor framework that future multivariate studies can build upon to identify independent determinants of AI adoption readiness in dental education.

Almost half (49.2%) of the students in the current study gained knowledge about AI from their teachers, followed by other formal resources such as lectures, seminars, and workshops, contradicting previous studies from Saudi Arabia in which participants reported that they mainly gained knowledge from social media [22, 29]. Educators play a crucial role in AI instruction, therefore highlighting the need for faculty training and providing hands‐on experiences. Incorporating AI in dental education will transform knowledge acquisition and improve teaching strategies [3, 26, 37, 38]. Educators can use these findings to improve AI integration in education, especially in dental fields, which have been highlighted by Dhingra [39], Lingam et al. [40], and Chen et al. [41]. The pandemic introduced chatbots for research [42]. AI enhances diagnostic skills and helps with appointment scheduling and patient management [42]. These are some possible ways to incorporate AI into the dental curriculum, which may vary depending on the institution’s goals [26].

### 4.5. Enhancing Clinical Decision‐Making and Skills

The study found that junior students use AI more than senior students for personal tasks like planning and entertainment. Junior students are part of a more digitally native generation, familiar with AI‐powered applications, and have more time to explore AI for non‐academic purposes as they are less burdened by clinical responsibilities and final‐year demands. Pauwels and Del Rey [15] reported a positive attitude towards AI in oral radiology among dental professionals and students. Similarly, Yüzbaşıoğlu [16] found that 85% of students agreed that the use of AI would bring innovative changes in the field of dentistry, and 74% of respondents agreed on the addition of topics related to AI in the undergraduate curriculum. Future applications can include imaging analysis, analyzing dental records for improved diagnosis, and creating 3D models of patients’ jaws. AI can also monitor healing and recognize changes in treatment [43]. AI can personalize orthodontic treatments by predicting outcomes from past data [44, 45]. In periodontics, AI aids in diagnosing diseases [46, 47] and improves accuracy in restorative dentistry [40]. AI models were reported to detect oral cancer with 80% to 93% accuracy [48, 49].

AI applications in diagnostics, imaging, and treatment planning offer opportunities to enhance students’ clinical reasoning skills. For example, AI systems have been reported to improve diagnostic accuracy and assist in treatment planning [40, 41]. Incorporating these tools into preclinical and clinical training can help students interpret AI‐generated outputs and develop critical‐thinking skills. This approach ensures that students are prepared to work alongside AI systems while maintaining professional judgment. Incorporating these tools into preclinical and clinical training can help students better understand diagnostic workflows, interpret AI‐generated outputs, and develop critical thinking when comparing AI‐assisted decisions with traditional clinical judgments. This approach not only enhances learning but also prepares students to work alongside AI systems in a safe and effective manner.

### 4.6. Ethical and Professional Considerations

Ethical concerns regarding AI use were reported by 53.4% of participants, with significantly higher concern among faculty (*p* = 0.007). These concerns include issues related to academic dishonesty, data privacy, and over‐reliance on AI systems. Similar concerns have been reported in the literature, emphasizing the need for clear ethical guidelines in AI use [8, 33]. Incorporating ethical training into dental education is essential to ensure responsible use of AI and to reinforce the importance of human oversight in clinical decision‐making.

The integration of AI into dental education is essential for preparing future professionals to work in a rapidly evolving healthcare environment. AI applications such as diagnostic support, patient management, and personalized treatment planning are expected to play a major role in future dental practices [43, 44]. By embedding AI into curricula, institutions can ensure that graduates are equipped with the skills necessary to effectively utilize these technologies, ultimately improving patient care and clinical outcomes. Given the concerns raised by both students and faculty regarding ethical implications, dental education must also emphasize the responsible use of AI. Topics such as data privacy, academic integrity, transparency, and limitations of AI systems should be incorporated into teaching. Preparing students to navigate these challenges is critical to ensuring that AI is used as a supportive tool rather than a substitute for professional judgment.

Ultimately, aligning dental education with advancements in AI will help prepare future dental professionals for a rapidly evolving healthcare landscape. By embedding AI into teaching and learning processes, institutions can ensure that graduates are not only aware of technological innovations but are also competent in integrating them into patient care. This alignment reinforces the relevance of AI in education and strengthens the overall impact of its adoption in dentistry.

Ethical concerns about the use of AI were perceived by students and faculty members, with statistically significantly higher concerns reported by faculty (*p* = 0.007). Faculty members often express concerns regarding ethical issues, such as excessive dependance on AI, risks to confidentiality, and the possibility of academic dishonesty. A qualitative research study revealed concerns among faculty regarding the potential misuse of generative AI tools by students in educational assessments, emphasizing the importance of establishing ethical guidelines for the use of AI in academic environments [33].

## 5. Limitations

The current study has some limitations. First, the study is from a single dental school in the eastern province of Saudi Arabia, which might affect the generalizability of its findings to other countries or educational settings; therefore, the data should be interpreted in the context of an exploratory, institution‐specific analysis rather than as broadly generalized conclusions. Second, the data was self‐reported, which may introduce bias due to over or underestimation. Also, the questionnaire lacked randomization of the item order, which would have further strengthened the study design. The study also focused on academic AI tools, limiting the exploration of AI in clinical practice, and although ethical concerns were explored, they were not deeply examined. There is a need for qualitative data for more detailed insights as well as longitudinal follow‐up studies to evaluate the impact of training on AI knowledge and ethics awareness. As the data presented are based on self‐reported responses from students and faculty of a single institution, future follow‐up studies must be conducted while involving multiple institutions with a large sample size to produce data which can provide a more generalized outcome. Additionally, the absence of multivariate regression modeling limits the identification of independent predictors of AI adoption; future studies should employ ordinal logistic regression or structural equation modeling to disentangle the relative contributions of role, gender, academic level, and prior AI exposure to adoption readiness outcomes. Furthermore, the cross‐sectional design precludes causal inference, and self‐report bias may affect the accuracy of AI knowledge and usage estimates.

## 6. Future Directions

Future research should prioritize (1) multi‐institution replication across diverse Saudi and regional contexts to enhance generalizability; (2) longitudinal designs to track AI literacy and adoption trajectories across the dental training continuum; (3) multivariate modeling (ordinal logistic regression and structural equation modeling) to identify independent predictors of AI curriculum support; and (4) qualitative inquiry to explore the motivational and structural barriers that underlie the knowing‐doing gap observed between faculty knowledge and practice.

## 7. Conclusion

This study found that dental students and faculty demonstrated moderate awareness of AI tools, with generally favorable perceptions regarding their relevance to dental education. Female students reported greater use of AI tools for academic tasks, while male students showed marginally higher self‐assessed knowledge of AI concepts. Undergraduates were more familiar with specific AI tools than postgraduates and interns. Faculty members reported greater AI knowledge and more ethical concerns compared to students, and both groups expressed support for incorporating AI‐related training into the dental curriculum.

## Funding

No funding was received for this manuscript.

## Conflicts of Interest

The authors declare no conflicts of interest.

## Data Availability

The data that support the findings of this study are available from the corresponding author upon reasonable request.
